# Characterization of a novel recombinant halophilic β-glucosidase of *Trichoderma harzianum* derived from Hainan mangrove

**DOI:** 10.1186/s12866-022-02596-w

**Published:** 2022-07-28

**Authors:** Nan Sun, Xiaoxuan Liu, Bingxi Zhang, Xuemei Wang, Wei Na, Zhen Tan, Xiaochun Li, Qingfeng Guan

**Affiliations:** 1 Lab of Animal Nutrition, Reproduction & Breeding, College of Animal Science and Technology, Hainan University, No.58 Renmin Avenue, Meilan, Haikou, 570228 P. R. China; 2 Lab of Animal Genetics, Reproduction & Breeding, College of Animal Science and Technology, Hainan University, No.58 Renmin Avenue, Meilan, Haikou, 570228 P. R. China; 3 Lab of Microorganism Resource and Utilization Research, School of Life Sciences, Hainan University, No.58 Renmin Avenue, Meilan, Haikou, 570228 P. R. China

**Keywords:** *Trichoderma harzianum*, β-glucosidase, Thermal stability, Salt tolerance

## Abstract

**Background:**

β-glucosidase is an important biomass-degrading enzyme and plays a vital role in generating renewable biofuels through enzymatic saccharification. In this study, we analyzed the transcriptome of *Trichoderma harzianum* HTASA derived from Hainan mangrove and identified a new gene encoding β-glucosidase Bgl3HB. And the biochemically characterization of β-glucosidase activity was performed.

**Results:**

Bgl3HB showed substantial catalytic activity in the pH range of 3.0–5.0 and at temperatures of 40 ℃-60 ℃. The enzyme was found quite stable at 50 ℃ with a loss of only 33.4% relative activity after 240 min of heat exposure. In addition, all tested metal ions were found to promote the enzyme activity. The β-glucosidase activity of Bgl3HB was enhanced by 2.12-fold of its original activity in the presence of 5 M NaCl. Surprisingly, Bgl3HB also showed a remarkable ability to hydrolyze laminarin compared to other measured substrates. Enzyme efficiency was examined in the sugarcane bagasse saccharification processes, in which Bgl3HB with 5 M NaCl worked better supplementing Celluclast 1.5L than the commercial Novozyme 188 ascertained it as an admirably suited biocatalyst for the utilization of agricultural waste. In this work, this is the first report of a halophilic β-glucosidase from *Trichoderma harzianum*, and represents the β-glucosidase with the highest known NaCl activation concentration. And adding 5 M NaCl could enhance saccharification performance even better than commercial cellulase.

**Conclusions:**

These results show that Bgl3HB has great promise as a highly stable and highly efficient cellulase with important future applications in the industrial production of biofuels.

**Supplementary Information:**

The online version contains supplementary material available at 10.1186/s12866-022-02596-w.

## Background

Plant biomass is the most abundant and widely distributed renewable biological resource in the world, about two-thirds of which consists of cellulose and hemicellulose. The biodegradation of cellulosic materials has been reported to have potential importance in a variety of industrial and agricultural applications [1, 2]. The complete hydrolysis of cellulose to glucose requires the combined actions of endoglucanases (EC 3.2.1.4, EG) and exoglucanases (EC 3.2.1.91, CBH I and CBH II), as well as β-glucosidases (EC 3.2.1.21, BGL) [3]. EG catalyzes the breakdown of internal β-1, 4-linkages at random positions on the glucose polymer chain, while CBH mainly cleaves the cellobiose residues from the terminal end (CBH I and CBH II cleave from the reducing and non-reducing terminal, respectively) [4, 5]. The resulting cellobiose or cellooligosaccharide is hydrolyzed from the non-reducing terminal to a single unit of glucose by BGL, thereby releasing glucose, which helps reduce the product inhibition of cellobiose on EG and CBH [6]. In particular, for the complete saccharification of cellulosic materials, β-glucosidases were identified as key enzymes representing the major bottleneck of efficient hydrolysis [7]. Especially, the formation of end products (i.e., glucose and xylose) and other chemical agents (i.e., salt and ionic solutions) could inhibit β-glucosidase activity and greatly limit its industrial applications [8, 9]. Most β-glucosidases currently available on the market are sensitive to high glucose concentrations, with inhibitor constant (*K*_i_) values ranging from 0.5 mM to 100 mM [10]. Typically, *K*_i_ values greater than 200 mM indicate that most β-glucosidase activity will be inhibited during the saccharification of cellulose to glucose [11]. Temperature also affect the saccharification process. For example, Teugjas and Väljamäe [9] reported that increasing the temperature improves catalytic efficiency and, moreover, relieves product inhibition of endoglucanases and β-glucosidases. Thus, high hydrolytic efficiency and tolerance to unfavorable conditions are prerequisites of an industrial biocatalyst in various applications.

Mangroves are wetland ecosystems dominated by salt-tolerant woody plants, found in tropical and subtropical coastal areas. Their soil is often slightly acidic [12], and contain rich microbial resources, with community structure and diversity differing significantly across areas [1]. Studies characterizing mangrove soil have found that the microbial communities are dominated by bacteria, followed by actinomycetes and filamentous fungi. Filamentous fungi have been used in the commercial production of β-glucosidase due to their ability to utilize cheaper substrates as energy and carbon sources, thereby reducing the cost of industrial fermentation processes [13, 14]. *T. harzianum* is a common filamentous fungus. Studies on various *T. harzianum* strains showed that they can produce cellulolytic enzyme complex with higher β-glucosidase and endoglucanases activities than that shown by *T. reesei* [15]. These microbes, which can survive in extreme environments, are a key source of enzymes for use in heterogeneous biomass conversion processes, since they are equipped with genes that help them to thrive under extreme conditions [16]. Therefore, enzymes derived from mangrove may offer novel biocatalyst properties like high salt tolerance, metal tolerance, thermostability and cold adaptivity [17]. Previously, fungal metabolites with special functions have been reported in mangrove soil [18, 19], but mangrove-derived cellulase producers are not well described. Such novel filamentous fungal strains from mangroves hold great promise for industrial applications of cellulase production and biomass degradation.

The present study screened a fungus in mangrove seawater in Haikou City, Hainan Province, China. The fungus can grow under extremely acidic conditions. Acid β-glucosidase is very important in the food and beverage industry and the production of fuel ethanol. Therefore, in order to mine the high-quality enzyme resources of the fungus, we used different carbon sources as the only carbon sources, and obtained the differentially expressed gene bgl3HB of the fungus under acidic conditions by the comparative transcriptome, and obtained the Bgl3HB by exogenous expression of *Komagataella phaffii*. And we found that Bgl3HB is resistant to salt, acid and heat, and has high hydrolysis activity on laminabiose, so this novel β-glucosidase has great potential as a biocatalyst in several industrial applications.

## Results and discussion

### Gene mining and sequence analysis

Under the screening condition of FDR < 0.05 and |log2FC|> 2, a total of 756 Unigenes were selected. The gene sequences from assembled transcriptome data against the NR database resulted in the identification of β-glucosidase genes, *bgl3HB*, under the stringent criteria of at least 50% identity and 90% subject coverage. This putative β-glucosidase gene, in turn, did not reveal similarity with any of the sequences available in NCBI non-redundant nucleotide database on the default BLASTn parameters. This establishes the novelty of the β-glucosidase gene characterized in this study. The molecular weight and theoretical isoelectric point of Bgl3HB was predicted to be 86.66 kDa and 5.35, respectively, by the ExPASy website (https://web.expasy.org/compute_pi/). The SignalP5.0 and TMHMM2.0 analyses showed that the gene sequence of Bgl3HB had no signal peptide and belonged to an exocrine protein.

**Fig. 1:**
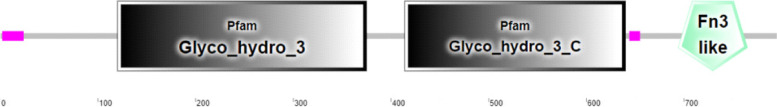
Domain analysis of Bgl3HB

The domains of Bgl3HB were analyzed with the SMART website (SMART: Main page (embl-heidelberg.de)). As shown in Fig. 1, Bgl3HB contains a Pfam Glyco-hydro-3 domain, a Pfam Glyco-hydro-3-C domain and a Fn3_like domain. Glyco_hydro_3 and Glyco_hydro_3_C domains belong to the Glycoside hydrolase family 3 (GH3) and participate in catalysis. GH3 comprises enzymes with a number of known activities; β-glucosidase ( EC 3.2.1.21); β-xylosidase (EC 3.2.1.37); N-acetyl β-glucosaminidase (EC 3.2.1.52); glucan β-1,3-glucosidase (EC 3.2.1.58); cellodextrinase (EC 3.2.1.74) and exo-1,3–1,4-glucanase (EC 3.2.1). These enzymes are two-domain globular proteins that are N-glycosylated at three sites. This suggests that Bgl3HB belongs to the GH3 family, with a Fn3-like domain in the C-terminal. The specific function of Fn3-like domain is unknown, which may be related to the thermal stability of the protein.

### Expression and purification of Bgl3HB

**Fig. 2:**
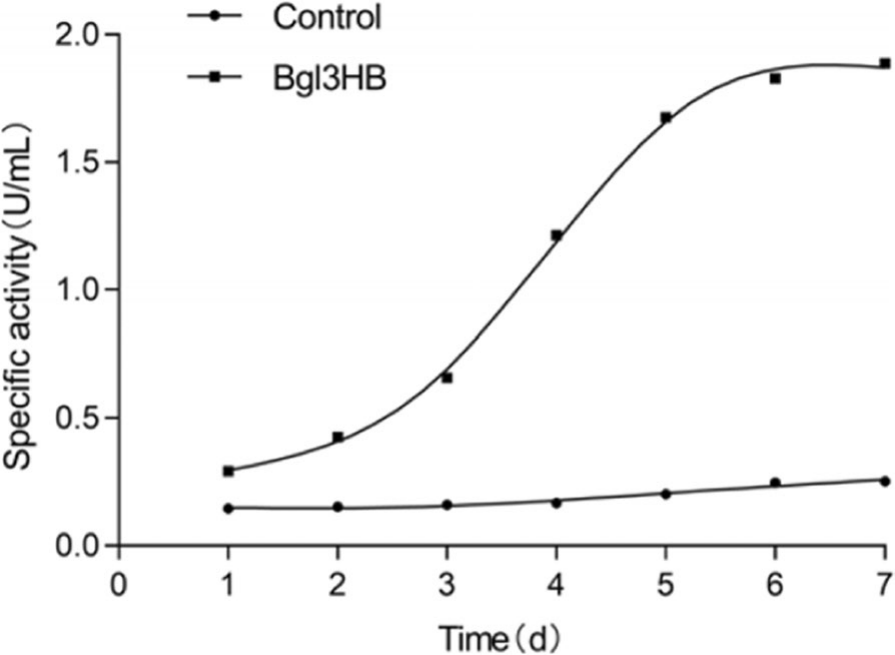
Changes in specific activity of Bgl3HB in fermentation supernatant for 7 days

**Fig. 3:**
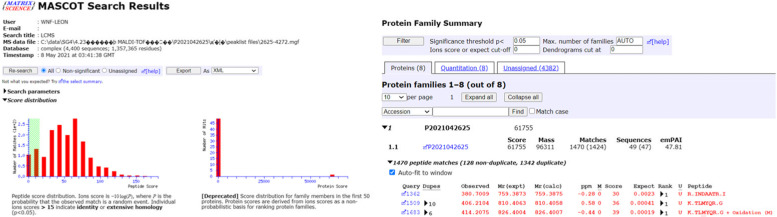
Identification of Bgl3HB

Recombinant Bgl3HB was successfully produced in *K. phaffii* X33 cultured cells in BMMY medium. Figure 2 showed the changes of the enzyme specific activity of the supernatant of the recombinant strain and the control strain under the induction of 1% methanol for 7 days. The mass spectrometry identification results are shown in Fig. 3.

**Fig. 4:**
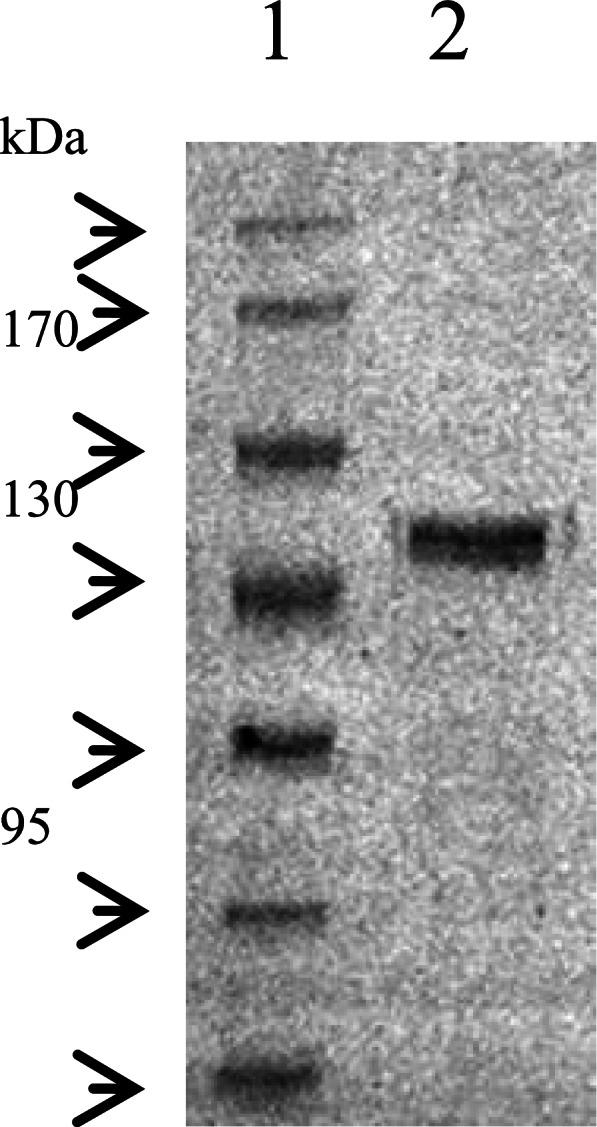
The molecular weight and purity of Bgl3HB were confirmed by SDS-PAGE on an 8% SDS gel. Lane 1: PageRuler Prestained Protein Ladder (Thermo Scientific, USA); and Lane 2: purified Bgl3HB stained by Coomassie Brilliant Blue BL605A

From the position of the band, it can be judged that Bgl3HB is correctly expressed (Fig. 4), given the similarity to the expected protein molecular weight.

### Biochemical characteristics of recombinant Bgl3HB

The enzymatic properties of purified recombinant Bgl3HB produced in *K. phaffii* X33 were determined using pNPG as the substrate.

**Fig. 5:**
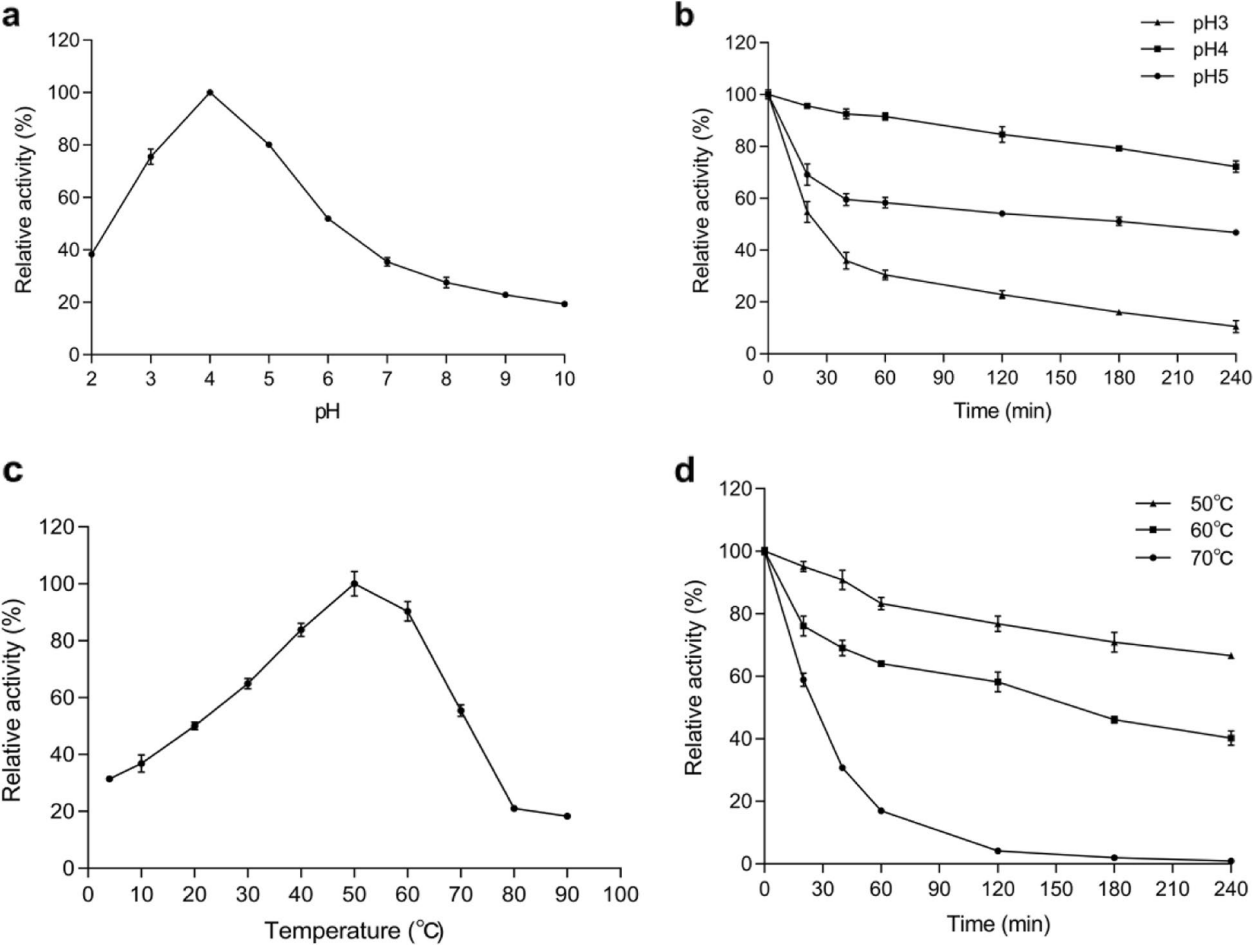
Enzymatic properties of the purified recombinant Bgl3HB produced in *K. phaffii* using pNPG as the substrate. **a** Effect of pH on enzyme activities. **b** pH stability of Bgl3HB after 240 min incubation at 4 °C. **c** Effect of temperature on enzyme activities. **d** Thermostability of Bgl3HB at pH 4.0 and different temperatures between 50 °C and 70 °C up to 240 min. Each value in the panel represents the mean ± SD (*n* = 3)

Purified Bgl3HB was active over a wide range of acidic pH, exhibiting optimal activity at pH 4.0 and retaining > 76% activity at pH 3.0–5.0 (Fig. 5a). The pH stability was investigated by measuring the residual activity after incubation at 4 °C, at pH values ranging from 3.0 to 5.0. As shown in Fig. 5b, Bgl3HB was stable at pH 4; it retained more than 73% of its residual activity after 240 min of incubation in pH buffer at 4 °C. Most fungal β-glucosidase show pH optima ranging from 4.0 to 6.5 and are usually stable over a wide pH range [20, 21]. This optimum pH and stability indicate that Bgl3HB have potential commercial value, because stability for a long time under extreme pH conditions is one of the most desired industrial properties of β-glucosidase. β-glucosidase that can tolerate low pH and remain relatively stable are very advantageous [22] in the use of pretreated lignocellulose slurry, which is usually acidic. In practical production applications, such as in the wine-making process, acid-resistant β-glucosidase plays a key role in the enzymatic release of aromatic compounds from glycoside precursors present in fruit juices and wines [23]. Acid-tolerant β-glucosidase is very important in the food and beverage industry and in the production of fuel ethanol from cellulosic materials [24].

The optimum temperature of the hydrolysis reaction was found to be 50 °C (Fig. 5c). When Bgl3HB activity was assayed at pH 4.0, it exhibited maximum activity at 50 °C and retained 90% activity even at 60 °C (Fig. 5c). Bgl3HB was also highly stable at 50 °C, retaining > 65% activity after 240 min (Fig. 5d). Previous results of *Aspergillus fumigatus* Z5 [25], *Trichoderma oranges* [26] and *Trichoderma mange* [27] showed that thermal decomposition is usually used to promote the degradation of lignocellulose biomass, so heat resistance is an ideal property of β-glucosidase. And thermostable β-glucosidases offer several advantages in industrial applications, such as promoting faster reactions, high solubility of the substrate and a lower risk of contamination, which has led to an increased desire for a new type of β-glucosidase with high reaction temperature and thermal stability [28]. In this study, Bgl3HB can even maintain more than 50% enzyme activity after incubation at 60 ℃ for 120 min. Thermostable β-glucosidase not only plays an extremely important role in the high temperature growth of *T. harzianum* in its native mangrove wetland, but also thermostable β-glucosidase in industrial processes such as biorefining. If the substrate needs to be heat treated, thermophilic enzymes can increase the reaction rate, shorten the hydrolysis time, and have excellent specific activity of reducing the number of enzymes. Higher temperature stability and better pH flexibility generally related to process configuration [29, 30]. Because most commercial cellulases currently on the market do not have good thermal stability, and β-glucosidase is the rate-limiting enzyme in the process of cellulose hydrolysis, the demand for thermostable β-glucosidase is significant. First, the temperature of enzymatic hydrolysis of plant biomass is 30–50 ℃, so a thermally stable β-glucosidase is favorable in this enzymatic reaction. Second, enzymatic hydrolysis at higher temperatures can effectively avoid contamination of the reaction with undesirable bacteria. Therefore, the β-glucosidase produced by *T. harzianum* from Hainan mangrove in our laboratory has a wide range of commercial value and application prospects.

### Effect of metal ions on enzymatic activity

**Fig. 6:**
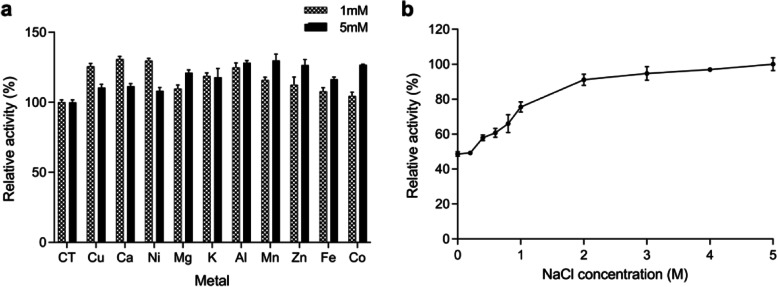
Effect of metals and NaCl on Bgl3HB enzyme activity with p-nitrophenyl-β-D-glucopyranoside (pNPG) as substrate. The values represent the mean ± SD (*n* = 3). **a** The reactions were performed by incubating purified enzyme with 1 mM and 5 mM of various metal ions (Cu^2+^, Ca^2+^, Ni^2+^, Mg^2+^, K^+^, Al^3+^, Mn^2+^, Zn^+^, Fe^3+^, Co^2+^) at 50 °C for 10 min; CT means the contrast group. **b** Enzyme was incubated in 0–5 M NaCl at 50 °C for 10 min and determined the specific activity of the enzyme. The highest enzyme specific activity was taken as equivalent to 100% specific activity

Bgl3HB was highly resistant to all tested 1 mM and 5 mM metal ions (Fig. 6a), and no similar reports had been found. In particular, Ca^2+^ and Mn^2+^ extremely enhanced Bgl3HB activity by 30.96% and 29.98%, respectively. Collectively, these results indicate that most metal ions can promote the activity of Bgl3HB, which meets the needs of industrial production. In the process of cellulose saccharification, metal ions often inhibit the activity of glucosidase, so metal ions tolerance is a desirable feature for enzymatic bioprocesses [31].

### Effect of NaCl on enzyme activity

The effects of salt on the activity of Bgl3HB were determined at the concentrations of 0–5 mM (Fig. 6b). Notably, at higher concentrations of salt there was a stronger promotion of enzyme activity. To evaluate the activation level of Bgl3HB by NaCl compared to other β-glucosidases, we provide a summary of reported β-glucosidases that can be activated by NaCl in Table 1. Bgl3HB is the β-glucosidase exhibiting the highest NaCl activation concentration, and it is the first halophilic β-glucosidase from *T. harzianum* to be reported. This may be because mangroves are marine ecosystems, with high salinity and soil osmotic pressure, leading to salt stress tolerance of most microorganisms [17]. β-glucosidase isolated from mangrove soil has previously been shown to be salt tolerant, and enzyme activity can even be enhanced by salt concentrations. Most of the β-glucosidases isolated from oceanic microbes show similar properties. It has been reported that a cellulase produced by marine *Aspergillus niger* ZJUBE-1 isolated from the sludge of the East China Sea can retain nearly 80% of the enzyme activity in the presence of 4.5 M NaCl [32]. The β-glucosidase produced by *Streptomyces* from deep-sea sediments can stably exist in 1 M NaCl for 48 h [17]. In this study, Bgl3HB derived from mangroves was shown to tolerate NaCl up to 5 M (higher than seawater salinity), and its activity increased by 2 times. Microbes living in mangroves often have to cope with extreme salinity gradients caused by seasonal rainfall, tidal conditions and freshwater outflows [33]. In addition, the distribution of species is regulated by high, medium and low salinity regions, which can explain the tolerance of Bgl3HB to NaCl.

**Table 1:** Comparison of activity toward pNPG by β-glucosidases activated by NaCl

Enzyme origin	Optimal NaCl concentration (M)	Highest activation multiple of enzyme activity	Reference
*Pseudoalteromonas sp.GXQ-1*	3	8.74-Fold	[34]
*Thermococcus sp.*	1.5	1.24-Fold	[16]
*Streptomycete*	0.5	1.62-Fold	[17] [2]
*Aspergillus niger*	4	1.44-Fold	[35]
*Bacillus sp. SJ-10*	2.57	3.36-Fold	[36]
*Trichoderma harzianum HTASA*	5	2.12-Fold	This study

### Substrate specificity assays and kinetic parameters of Bgl3HB

To determine substrate specificity, the hydrolytic activity of purified Bgl3HB was measured using various substrates. We found that Bgl3HB showed extensive hydrolytic activity against many substrates. It had high specific activity of 58.34 U/mg on daidzein and 129.79 U/mg on laminarin. When using disaccharides of different linkages as the substrate, the enzymes showed different levels of preference, in the order of Gentiobiose (β-1,6 linkage) > Sophorose (β-1,2 linkage) > Laminaribiose (β-1,3 linkage) > Cellobiose (β-1,4 linkage) > Cellopentaose (β-1,4 linkage) > Cellotetraose (β-1,4 linkage) > Cellotriose (β-1,4 linkage) (Table 2). And for various aryl-glycoside substrates, Bgl3HB was active with daidzein and laminarin as preferred substrates. Surprisingly, it also showed a remarkable ability to hydrolyze laminarin, a β-1,3-glucan present in algae, which makes Bgl3HB a good candidate for using in the production of 3G biofuels from algal biomass [37]. Recently, numerous algae species have been studied for this objective. For example, green algae, including *Spirogyra sp.* and *Chlorococcum sp.*, accumulate high levels of polysaccharides like starch, which could be fermented to bioethanol [38]. Méndez-Líter found a β-glucosidase from the ascomycete fungus *Talaromyces amestolkiae*, which had a higher hydrolysis rate than a commercial laminarinase for laminarin [37].

**Table 2:** Substrate specificity of purified recombinant β-glucosidase Bgl3HB

Substrates	Specific activity (U/mg)
**Disaccharides**	
Cellobiose	5.38 ± 0.027
Cellotriose	3.61 ± 0.064
Cellotetraose	3.91 ± 0.456
Cellopentaose	4.20 ± 0.347
Sophorose	6.55 ± 0.205
Gentiobiose	8.33 ± 0.392
Laminaribiose	6.40 ± 0.059
**Aryl-glycosides**	
Daidzein	58.34 ± 0.014
Laminarin	129.79 ± 0.415
pNPG	52.63 ± 0.132

^a^ Data is shown as mean ± standard deviation (*n* = 2)

The kinetics of Bgl3HB on daidzein, laminarin and pNPG are shown in Table 3. Bgl3HB exhibited much higher substrate affinity and catalytic efficiency on laminarin than those of other Aryl-glycosides. It had the lowest *KM* value, which means that Bgl3HB had high affinity for laminarin. The versatility of some β-glucosidases to hydrolyze short oligosaccharides with β-1,2, β-1,3, β-1,4, or β-1,6 linkages has been widely reported [39]. However, the ability of Bgl3HB to degrade a polysaccharide like laminarin is truly exceptional, which makes Bgl3HB have great advantages in the production of biofuel with algae biomass as raw material. Recently, 3G biofuels derived from algae biomass have received considerable attention for their advantages as biodiesel, bioethanol, biohydrogen, and biomethane [40]. This catalytic property of Bgl3HB could be of great interest for depolymerization of glucans for 3G bioethanol production.

**Table 3:** Kinetic parameters of Bgl3HB with different substrates

Substrates	*K*M(mM)	*V*_max_(μmol/min/mg)	*K*_cat_(s^−1^)	*K*_cat_*/K*M ((mM^−1^ s^−1^)
*p*NPG	1.22	112.03	989.20	810.06
Daidzein	1.04	70.37	621.37	594.99
Laminarin	0.61	69.25	611.47	1006.68

### Enzymatic saccharification of cellulose materials

**Fig. 7:**
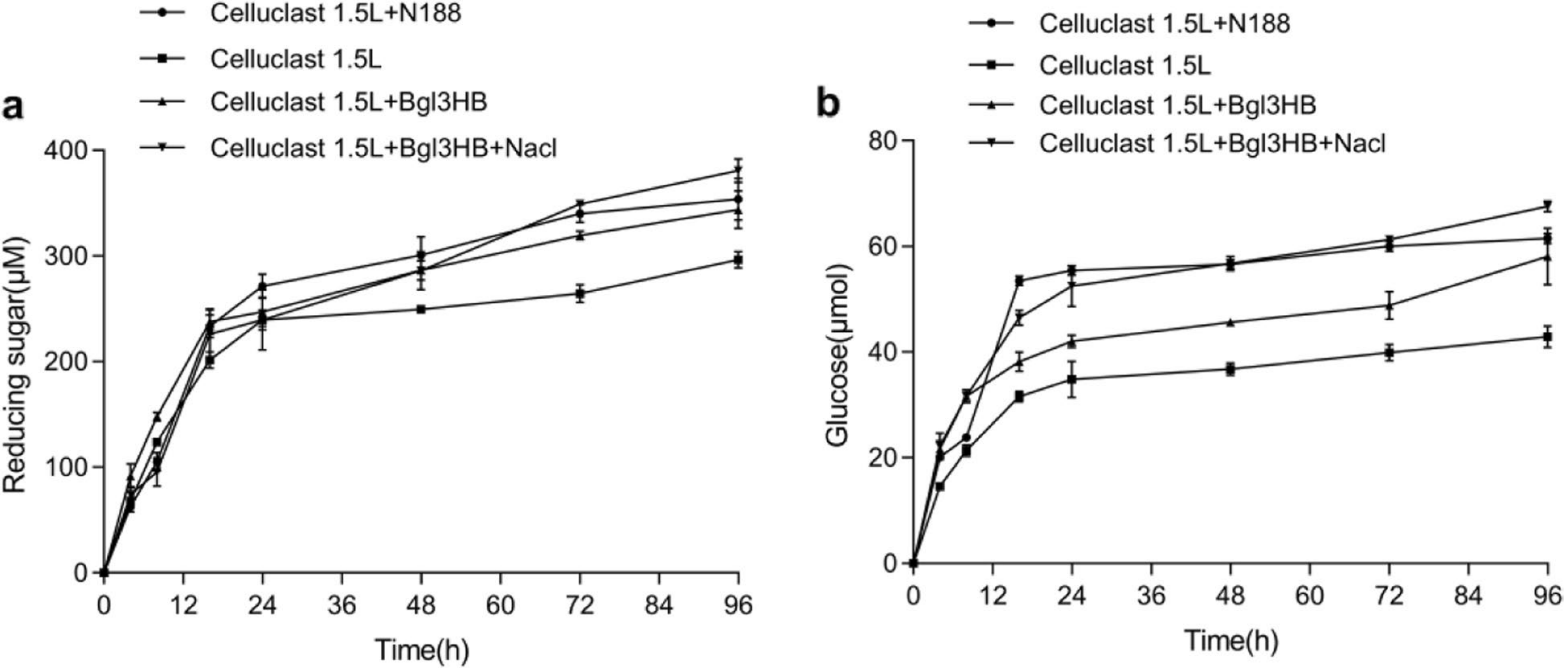
Enzymatic saccharification of Bgl3HB (12 BGU/g dry material) in combination with commercial cellulase (5 FPU/g dry material). The pretreated bagasse was used as the substrate. **a** The reducing sugar released by enzyme(s). **b** The glucose released by enzyme(s)

In order to evaluate the application value of Bgl3HB under practical conditions, the application potential of Bgl3HB in saccharification of cellulose raw materials was compared with that of commercial Novozyme 188 at pH 4.0 and 50 ℃. As shown in Fig. 7, bagasse was used as raw material, in the blank control group, commercial Celluclast 1.5L (5 FPU (Determined by pre-experiment)/g dry material) was cultured at pH 4.0, 50 °C for 96 h, reducing sugars from bagasse released 296.4 μmol, of which glucose accounted for 42.9 μmol (the glucose conversion rate of 14.5%). When β-glucosidase was added at 12 BGU (Determined by pre-experiment)/g dry material, Bgl3HB and commercial Novozyme 188 showed different synergistic effects in promoting saccharification. The synergistic action of Celluclast 1.5L and commercial Novozyme 188 released 353.8 μmol of reducing sugar and 61.5 μmol of glucose (glucose conversion rate 17.4%). Bgl3HB in combination with Celluclast 1.5L only released 344.0 μmol of reducing sugars and 58.1 μmol of glucose (the glucose conversion rate of 16.9%). However, Celluclast 1.5L and commercial Novozyme 188 with additional 5 mM NaCl improved performance in synergistic enzymatic saccharification. The yields of reducing sugars and fermentable glucose were 380.84 μmol and 67.60 μmol (the glucose conversion rate of 17.8%), which is higher than that of commercial Novozyme 188.

Notably, although the bagasse conversion rate of Bgl3HB is lower than that of commercial Novozyme 188, the glucose conversion rate is higher in Bgl3HB than in commercial Novozyme 188. However, the bagasse conversion rate of Bgl3HB with an additional 5 M NaCl was higher than that of commercial Novozyme 188, and the glucose conversion rate was also higher than that of commercial Novozyme 188. This agrees with other results in our study, suggesting that NaCl can increase Bgl3HB activity, and may help improve the hydrolysis capacity of Bgl3HB in the saccharification process. Such an effect may be because salt is conducive to the stability of the enzyme [16]. Qu et al. found that 3 M NaCl could improve the conversion rate of soybean isoflavones [34], which is similar to the findings of this paper. This important interaction would allow the hydrolysis of commercial cellulase with a smaller quantity of enzyme, which would reduce the industrial application cost.

### Conclusions

Here, a β-glucosidase gene derived from *T. harzianum* HTASA was cloned and heterologously expressed in *K. phaffii* X33. To our knowledge, this is the first report of a β-glucosidase identified from *T. harzianum* HTASA. A detailed enzymatic characterization of Bgl3HB was then performed. Bgl3HB was found to be heat-resisted and salt-tolerant, and could hydrolyze efficiently laminarin, which indicated that Bgl3HB had important cellulosic biomass degradation. Collectively, Bgl3HB is a salt-enhanced β-glucosidase with broad pH stability, high thermostability, uninhibited properties by most metal ions, and broad substrate specificity, which makes it has potential applications in different biotechnological applications, including bagasse hydrolysis and bioethanol production, and provides a useful material for further research.

## Methods

### Gene mining and sequence analysis

The transcriptome sequence of *T. harzianum* HTASA from mangrove in Hainan was mined to identify a novel gene encoding β-gluosidase, *bgl3HB*. The experimental design used glucose as the fermentation substrate as the control group and microcrystalline cellulose as the fermentation substrate as the experimental group. The assembled transcriptome data was subjected to Blastx against the Carbohydrate Active enZYme (CAZy) database, at the criteria of FDR < 0.05, |log2FC|> 2 and further aligned against non-redundant (NR) NCBI nucleotide and protein databases [41]. Domains are regions with specific structures and independent functions in biological macromolecules [42].The conserved domains and signal peptide in the putative β-glucosidase (Bgl3HB) were analyzed using signal 5.0 (http://www.cbs.dtu.dk/services/SignalP/) and SMART (https://smart.embl-heidelberg.de/smart/set_mode.cgi?GENOMIC=1) [43].

### Gene cloning and protein expression

The full-length gene *bgl3HB* was obtained from *T. harzianum* HTASA genomic DNA. The complete ORF of *bgl3HB* was amplified via polymerase chain reaction (PCR) using primer pairs listed in Table 4 and with Phanta® Max Super-Fidelity DNA Polymerase (Vazyme, China). The *bgl3HB* gene was cloned in plasmid pPICZαA digested by EcoRI-HF and XbaI and verified by sequencing. The recombinant plasmid was named pPICZαA/*bgl3HB* and transferred to *K. phaffii* competent cell X33 for expression.

**Table 4:** List of primers used for PCR in the study

Primers	Sequence	Purpose
*bgl3HBF*	ATGGTGAACAACGCAGCC	*bgl3HB* ORF cDNA
*bgl3HBR*	ATGATGACATTGGGATACTTATTGA	*bgl3HB* ORF cDNA
*bgl3HBEF*	AGAGAGGCTGAAGCTGAATTCATGGTGAACAACGCAGCCC	*bgl3HB* expression
*bgl3HBER*	GAGATGAGTTTTTGTTCTAGATTAATGATGATGATGATGATGTGGAGTGGCATCCTGAATCTGC	*bgl3HB* expression

^a^ The primers were designed by Primer Primier 5.0

The recombinant cells were cultured in BMGY medium at 28 ℃ and 200 G with 50 μg·ml^−1^ ampicillin. When this culture system achieved an optical density (OD600) of 2–8, the recombinant cells were transferred to 1000 mL BMMY medium, with 1 mL methanol added to the medium to induce gene expression. The induced culture was incubated at 28 ℃ and 200 G.

The induced cells were centrifuged at 12,000 G for 30 min at 4 ℃, and the supernatant was then filtered through a 0.22 μm syringe filter and concentrated with a 50 kDa ultrafiltration tube. The concentrated liquid was loaded on a pre-equilibrated BioScale™ Mini Cartridge (BIO-RAD, USA) for chromatographic separation of Bgl3HB protein. The BioScale™ Mini Cartridge was balanced with 10 mM buffer A (Na_2_HPO_4_-citric acid, pH 7.0) and 10 mM buffer B (Na_2_HPO_4_-citric acid with 1 M NaCl, pH 7.0). After passing through the BioScale™ Mini Cartridge, the concentrated liquid was washed using 20% buffer B. The purified protein was stored in HEPES (pH 5.0) at 4 ℃. SDS-PAGE electrophoresis was used to assess the molecular weight and purity of the protein. The concentration of purified protein was measured by a BCA protein concentration determination kit (Beyotime, China). Image acquisition tool was Universal Hood II (BIO-RAD, USA) and image processing software package was Image Lab.

### Enzyme assay

One unit of enzymatic activity (U) was defined as the amount of β-glucosidase required to hydrolyze the substrate to produce 1 μmol of product per minute under the given reaction conditions. Each assay was run in triplicate. The assay methods for different substrates were:

A. pNP glycoside substrates: At 50 °C and pH 4.0, 100 μL of 4 mM pNP glycoside substrate and 100 μL of purified enzyme solution were added to the reaction for 10 min, after which 600 μL of 1 M Na_2_CO_3_ was added to terminate the reaction. After cooling to room temperature, the absorbance at OD_400_ was measured. The enzymatic activity was determined according to the release amount of pNP [44].
B. Reducing oligosaccharide substrates. The GOD-POD method was used [45]. The 200 μL glucose Oxidase/Peroxidase Reagent was reacted with 100 μL of purified enzyme solution at 37 °C and pH 4.0 for 30 min, and 6 M H_2_SO_4_ was added to terminate the reaction. After cooling to room temperature, the absorbance at OD540 was measured. The enzyme activity was determined according to the amount of released glucose.
C. Polysaccharide substrates. The DNS method was used [46]. Preheated at 50 °C for 2 min with 900 μL of 0.5% (w/v) substrate solution, and then added 100 μL purified enzyme solution. After incubation at 50 °C and pH 4.0 for 10 min, 1.5 mL DNS was added and boiled for 5 min to terminate the reaction. The OD540 value was measured. The enzyme activity was determined according to the release amount of reducing sugar.

### Biochemical characterization

P-nitrophenyl-β-D-glucopyranoside (pNPG) was used as the substrate for biochemical characterization of purified recombinant Bgl3HB. The optimal temperature for Bgl3HB was determined at an optimal pH 4.0 for 10 min in the buffers with the temperatures ranging from 4–90 °C. The thermostability was determined by measuring the residual activity in buffer (50 mM Na_2_HPO_4_-citric acid) at various temperatures (50, 60, and 70 °C) for 0, 20, 40, 60, 120, 180 and 240 min [44]. The samples were rapidly cooled in an ice-water bath and residual activity was measured by the above method. The optimal pH for purified recombinant Bgl3HB was determined at 50℃ for 10 min in buffers with pH ranging from 2.0–10.0 (50 mM Na_2_HPO_4_-citric acid, pH 2.0–8.0; 50 mM glycine–NaOH pH 9.0–10.0). The pH stability of purified Bgl3HB was examined by measuring the residual enzyme activity under the standard conditions (50 °C for 10 min) at different pH values (pH 3.0–5.0) for 0, 20, 40, 60, 120, 180 and 240 min. The amount of residual activity was determined according to the activity assay method.

### Effect of metal ions and salt on enzymatic activity

To evaluate the effect of metal ions on enzymatic activity, 1 mM and 5 mM of various metal ions (Cu^2+^, Ca^2+^, Ni^2+^, Mg^2+^, K^+^, Al^3+^, Mn^2+^, Zn^+^, Fe^3+^, Co^2+^) under the standard conditions were added individually to the reaction system. The contrast group was tested using the same process described above without any additives to the reaction mixture. The effect of salt on Bgl3HB activity was tested by adding different concentrations of NaCl (0, 0.2 M, 0.4 M, 0.6 M, 0.8 M, 1 M, 2 M, 3 M, 4 M and 5 M) under the standard conditions (pH 4.0 and 50 °C for 10 min) [3].

### Substrate specificity and Kinetic constants

To determine the substrate specificity of Bgl3HB, the following 4 mM substrates were tested: cellobiose, cellotriose, cellotetraose, cellopentaose, gentiobiose, sophorose, laminaribiose, daidzein, laminarin and pNPG. For each, Bgl3HB activity was assayed under standard conditions (pH 4.0 and 50 °C for 10 min). All substrates were purchased from Shanghaiyuanye Bio-Technology (Shanghai, China). The enzyme inactivated after high temperature treatment was used as control, and the activity unit was U/mg.

The kinetic constants (*V*_*max*_, *KM*, *K*_*cat*_, and *K*_*cat*_/*KM*) of Bgl3HB were determined using different concentrations of daidzein, pNPG and laminarin (1 to 4 mM) at the optimal assay conditions. The value of *V*_*max*_, *KM*, *K*_*cat*_, and *K*_*cat*_/*KM* were calculated according to the Lineweaver–Burk plots, employing GraphPad prism 9.0.

### Sugarcane bagasse hydrolysis by Bgl3HB at 50 °C

Sugarcane bagasse was used as raw material for an enzymatic saccharification experiment to compare the saccharification efficiency of recombinant enzyme Bgl3HB and commercial Novozyme 188.

The specific steps are as follows: Sugarcane bagasse was pretreated with 1% NaOH at 121 °C for 30 min in an autoclave, washed with ddH_2_O and mixed with 20 mL of 100 mM Na_2_HPO_4_-citric acid buffer (pH 4.0) in 100 mL shake flasks at the concentration of 2% (dry material), while the corresponding enzyme components were added to each shake flask. Each enzyme combination was sampled at 4 h, 8 h, 12 h, 24 h, 48 h, 72 h and 96 h, and the reaction was terminated in boiling water bath. The amount of reducing sugars and glucose released in the supernatants were determined using the DNS [46] and GOD-POD methods, respectively. All these experiments were conducted with three replicates. The full experimental design is illustrated in Table 5. Additionally, the conversion of sugarcane bagasse was determined by adding 5 M NaCl.

**Table 5:** Enzymatic saccharification of Bgl3HB

Enzyme combination	Enzyme activity (/g)
Celluclast 1.5L (The control group)	5 FPU
Celluclast 1.5L + Novozyme 188	5 FPU, 12 BGU
Celluclast 1.5L + Bgl3HB	5 FPU, 12 BGU
Celluclast 1.5L + Bgl3HB + 5 M NaCl	5 FPU, 12 BGU

^a^ FPU: filter paper activity

^b^ BGU: β-glucosidase activity

## Supplementary Information

**Additional file 1.** 

**Additional file 2.** 

**Additional file 3.** 

**Additional file 4.** 

## Data Availability

All data generated or analysed during this study are included in this published article (and its supplementary information files).
