# Supplementary material for: Characterization of a novel recombinant halophilic β-glucosidase of Trichoderma harzianum derived from Hainan mangrove
Source: BMC Microbiol. 2022 Jul 28;22:185. doi: 10.1186/s12866-022-02596-w (PMC9331182; doi:10.1186/s12866-022-02596-w)
Supplement: Supplementary file 1 — Additional file 1. [file 12866_2022_2596_MOESM1_ESM.docx]

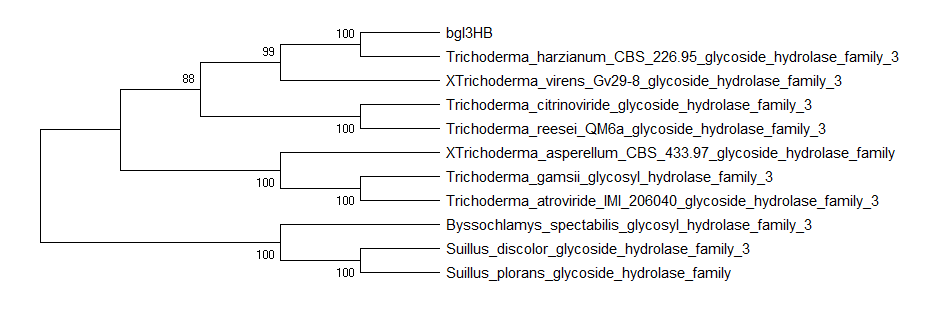


**Fig.1** Phylogenetic tree analysis comparing bgl3HB with other proteins with the GH3 family of the CAZy database

**
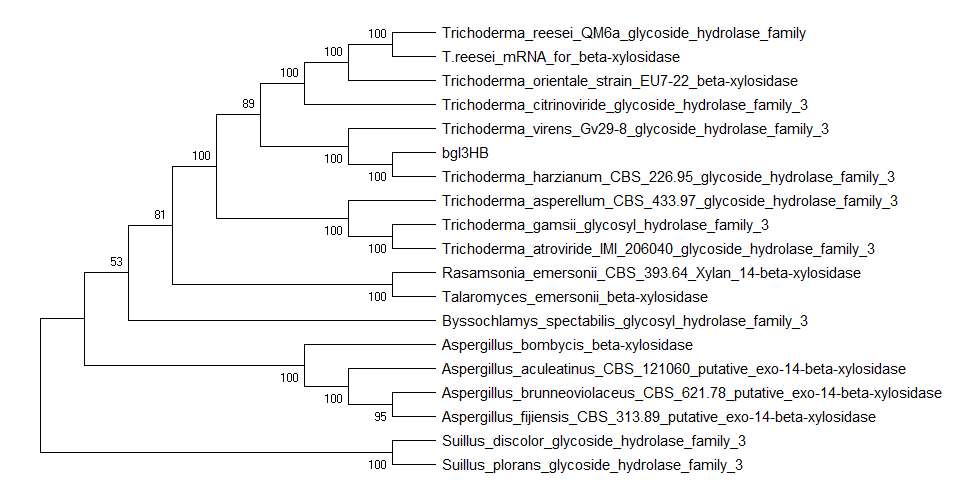
**

**Fig.2** Phylogenetic tree analysis of sequences with more than 70% similarity to bgl3HB sequences in NR database was performed


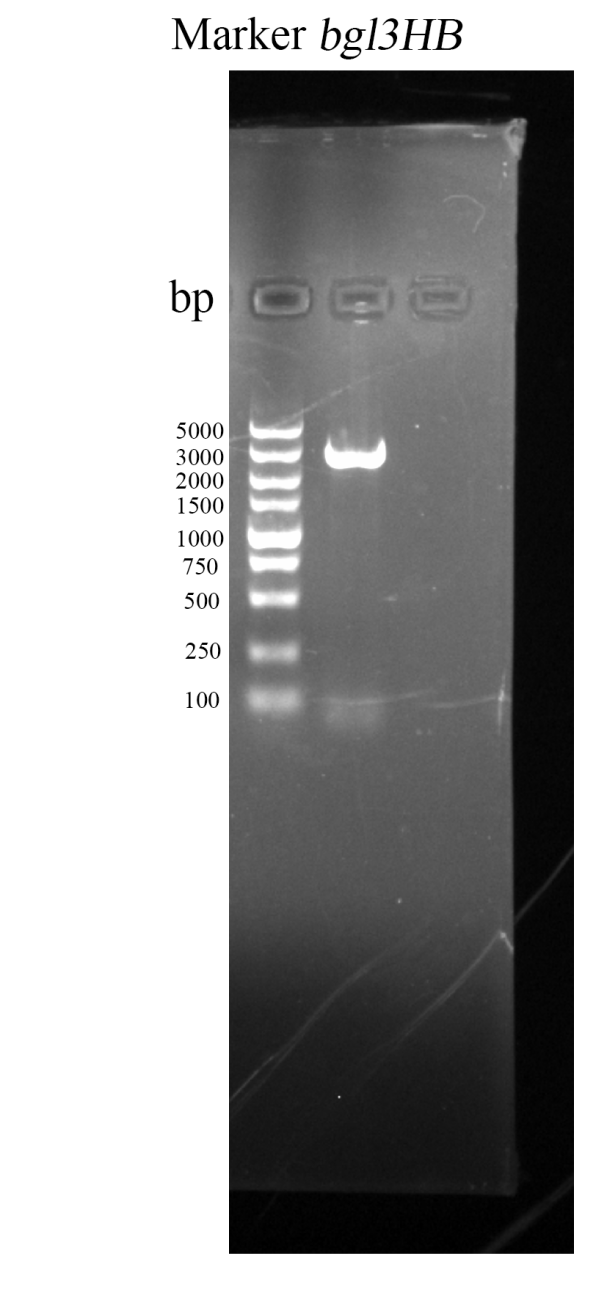


**Fig.3** Analysis of bgl3HB expression sequence of β-glucosidase gene by agarose gel electrophoresis

M: DNA maker，1: PCR-amplified bgl3HB gene (2430 bp)

**Table1** PCR reaction system

| ddH_2_O | 18μL |
| --- | --- |
| 2x Phanta Max Buffer | 25μL |
| dNTP | 1μL |
| Forward Primer | 2μL |
| Reverse Primer | 2μL |
| Phanta Max Super-fidelity DNA Polymerse | 1μL |
| Template | 1μL |

**Table 2** PCR reaction program

| Program | Temperature/℃ | Time/min | Cycle |
| --- | --- | --- | --- |
| Pre-denaturation | 95 | 3 | 1 |
| Denaturation | 95 | 0.25 | 3 |
| Annealing | 65 | 0.25 |  |
| Extend | 72 | 1.5 |  |
| Repair and extension | 72 | 5 | 1 |

**Table3** Recombination reaction system

| Component | Recombination reaction/μL | Negative control-1/μL | Negative control -2/μL | Positive control /μL |
| --- | --- | --- | --- | --- |
| Vector | 1 | 1 | 0 | 1 |
| Recombinant gene fragment | 3 | 0 | 3 | 1 |
| 5×CE II Buffer | 4 | 0 | 0 | 4 |
| Exnase II | 2 | 0 | 0 | 2 |
| ddH_2_O | 10 | 19 | 17 | 12 |

**Table4** Transformation of *Komagataella phaffii*

| Program | Time |
| --- | --- |
| Recombinant products and competent cells are mixed and reacted on ice | 30min |
| Heat shock at 42°C | 45s |
| Add 700mL liquid LB medium, shake at 37℃, 200G | 60min |
| The transformed competent cells were coated on LB medium | 8h |

**Table5** chromatographic procedure

| 1. Set pump flow rate to 6.0 mL/min (288 cm/hr) for the 5 mL cartridge. |
| --- |
| 2. Wash the cartridge with degassed low-salt buffer for 2 min. |
| 3. Wash the cartridge with degassed high-salt buffer for 5 min. |
| 4. Equilibrate the cartridge with low-salt buffer for 5 min. |
| 5. Equilibrate the cartridge with low-salt buffer for 5 min. |

Volume column: 5mL

Flux: 6.0 mL/min

Fraction volume: 5mL
