# Supplementary material for: Characterization of a novel recombinant halophilic β-glucosidase of Trichoderma harzianum derived from Hainan mangrove
Source: BMC Microbiol. 2022 Jul 28;22:185. doi: 10.1186/s12866-022-02596-w (PMC9331182; doi:10.1186/s12866-022-02596-w)
Supplement: Supplementary file 2 — Additional file 2. [file 12866_2022_2596_MOESM2_ESM.docx]

**1.*T. harzianum* ITS sequence (593bp)**

TGCGGAGGGATCATTACCGAGTTTACAACTCCCAAACCCAATGTGAACGTTACCAAACTGTTGCCTCGGCGGGATCTCTGCCCCGGGTGCGTCGCAGCCCCGGACCAAGGCGCCCGCCGGAGGACCAACCAAAACTCTTATTGTATACCCCCTCGCGGGTTTTTTTATAATCTGAGCCTTCTCGGCGCCTCTCGTAGGCGTTTCGAAAATGAATCAAAACTTTCAACAACGGATCTCTTGGTTCTGGCATCGATGAAGAACGCAGCGAAATGCGATAAGTAATGTGAATTGCAGAATTCAGTGAATCATCGAATCTTTGAACGCACATTGCGCCCGCCAGTATTCTGGCGGGCATGCCTGTCCGAGCGTCATTTCAACCCTCGAACCCCTCCGGGGGGTCGGCGTTGGGGATCGGCCCTCCCTTAGCGGGTGGCCGTCTCCGAAATACAGTGGCGGTCTCGCCGCAGCCTCTCCTGCGCAGTAGTTTGCACACTCGCATCGGGAGCGCGGCGCGTCCACAGCCGTTAAACACCCAACTTCTGAAATGTTGACCTCGGATCAGGTAGGAATACCCGCTGAACTTAAGCATATCAA

| **[Description](https://blast.ncbi.nlm.nih.gov/Blast.cgi)** | **[Max Score](https://blast.ncbi.nlm.nih.gov/Blast.cgi?CMD=Get&ADV_VIEW=yes&ADV_VIEW=on&ALIGNMENTS=10&ALIGNMENT_VIEW=Pairwise&CONFIG_DESCR=2,3,4,5,6,7,8&DATABASE_SORT=0&DESCRIPTIONS=10&DYNAMIC_FORMAT=on&FIRST_QUERY_NUM=0&FORMAT_NUM_ORG=1&FORMAT_OBJECT=Alignment&FORMAT_PAGE_TARGET=&FORMAT_TYPE=HTML&GET_SEQUENCE=yes&I_THRESH=&LINE_LENGTH=60&MASK_CHAR=2&MASK_COLOR=1&NUM_OVERVIEW=10&PAGE=MegaBlast&QUERY_INDEX=0&QUERY_NUMBER=0&RESULTS_PAGE_TARGET=&RID=FFYD4KZ7016&SHOW_LINKOUT=yes&SHOW_OVERVIEW=yes&STEP_NUMBER=&ADV_VIEW=on&DISPLAY_SORT=1&HSP_SORT=1)** | **[Total Score](https://blast.ncbi.nlm.nih.gov/Blast.cgi?CMD=Get&ADV_VIEW=yes&ADV_VIEW=on&ALIGNMENTS=10&ALIGNMENT_VIEW=Pairwise&CONFIG_DESCR=2,3,4,5,6,7,8&DATABASE_SORT=0&DESCRIPTIONS=10&DYNAMIC_FORMAT=on&FIRST_QUERY_NUM=0&FORMAT_NUM_ORG=1&FORMAT_OBJECT=Alignment&FORMAT_PAGE_TARGET=&FORMAT_TYPE=HTML&GET_SEQUENCE=yes&I_THRESH=&LINE_LENGTH=60&MASK_CHAR=2&MASK_COLOR=1&NUM_OVERVIEW=10&PAGE=MegaBlast&QUERY_INDEX=0&QUERY_NUMBER=0&RESULTS_PAGE_TARGET=&RID=FFYD4KZ7016&SHOW_LINKOUT=yes&SHOW_OVERVIEW=yes&STEP_NUMBER=&ADV_VIEW=on&DISPLAY_SORT=2&HSP_SORT=1)** | **[Query Cover](https://blast.ncbi.nlm.nih.gov/Blast.cgi?CMD=Get&ADV_VIEW=yes&ADV_VIEW=on&ALIGNMENTS=10&ALIGNMENT_VIEW=Pairwise&CONFIG_DESCR=2,3,4,5,6,7,8&DATABASE_SORT=0&DESCRIPTIONS=10&DYNAMIC_FORMAT=on&FIRST_QUERY_NUM=0&FORMAT_NUM_ORG=1&FORMAT_OBJECT=Alignment&FORMAT_PAGE_TARGET=&FORMAT_TYPE=HTML&GET_SEQUENCE=yes&I_THRESH=&LINE_LENGTH=60&MASK_CHAR=2&MASK_COLOR=1&NUM_OVERVIEW=10&PAGE=MegaBlast&QUERY_INDEX=0&QUERY_NUMBER=0&RESULTS_PAGE_TARGET=&RID=FFYD4KZ7016&SHOW_LINKOUT=yes&SHOW_OVERVIEW=yes&STEP_NUMBER=&ADV_VIEW=on&DISPLAY_SORT=4&HSP_SORT=0)** | **[E value](https://blast.ncbi.nlm.nih.gov/Blast.cgi?CMD=Get&ADV_VIEW=yes&ADV_VIEW=on&ALIGNMENTS=10&ALIGNMENT_VIEW=Pairwise&CONFIG_DESCR=2,3,4,5,6,7,8&DATABASE_SORT=0&DESCRIPTIONS=10&DYNAMIC_FORMAT=on&FIRST_QUERY_NUM=0&FORMAT_NUM_ORG=1&FORMAT_OBJECT=Alignment&FORMAT_PAGE_TARGET=&FORMAT_TYPE=HTML&GET_SEQUENCE=yes&I_THRESH=&LINE_LENGTH=60&MASK_CHAR=2&MASK_COLOR=1&NUM_OVERVIEW=10&PAGE=MegaBlast&QUERY_INDEX=0&QUERY_NUMBER=0&RESULTS_PAGE_TARGET=&RID=FFYD4KZ7016&SHOW_LINKOUT=yes&SHOW_OVERVIEW=yes&STEP_NUMBER=&ADV_VIEW=on&DISPLAY_SORT=0&HSP_SORT=0)** | **[Per. Ident](https://blast.ncbi.nlm.nih.gov/Blast.cgi?CMD=Get&ADV_VIEW=yes&ADV_VIEW=on&ALIGNMENTS=10&ALIGNMENT_VIEW=Pairwise&CONFIG_DESCR=2,3,4,5,6,7,8&DATABASE_SORT=0&DESCRIPTIONS=10&DYNAMIC_FORMAT=on&FIRST_QUERY_NUM=0&FORMAT_NUM_ORG=1&FORMAT_OBJECT=Alignment&FORMAT_PAGE_TARGET=&FORMAT_TYPE=HTML&GET_SEQUENCE=yes&I_THRESH=&LINE_LENGTH=60&MASK_CHAR=2&MASK_COLOR=1&NUM_OVERVIEW=10&PAGE=MegaBlast&QUERY_INDEX=0&QUERY_NUMBER=0&RESULTS_PAGE_TARGET=&RID=FFYD4KZ7016&SHOW_LINKOUT=yes&SHOW_OVERVIEW=yes&STEP_NUMBER=&ADV_VIEW=on&DISPLAY_SORT=3&HSP_SORT=3)** | **Accession** |
| --- | --- | --- | --- | --- | --- | --- |
| [Trichoderma harzianum isolate M3951 small subunit ribosomal RNA gene, partial sequence; internal transcribed spacer 1, 5.8S ribosomal RNA gene, and internal transcribed spacer 2, complete sequence; and large subunit ribosomal RNA gene, partial sequence](https://blast.ncbi.nlm.nih.gov/Blast.cgi#alnHdr_1606717380) | 1098 | 1098 | 100% | 0.0 | 100.00% | [MK738149.1](https://www.ncbi.nlm.nih.gov/nucleotide/MK738149.1?report=genbank&log$=nucltop&blast_rank=1&RID=FFYD4KZ7016) |
| [Trichoderma sp. isolate SDAS204116 small subunit ribosomal RNA gene, partial sequence; internal transcribed spacer 1, 5.8S ribosomal RNA gene, and internal transcribed spacer 2, complete sequence; and large subunit ribosomal RNA gene, partial sequence](https://blast.ncbi.nlm.nih.gov/Blast.cgi#alnHdr_1628768860) | 1098 | 1098 | 100% | 0.0 | 100.00% | [MK870785.1](https://www.ncbi.nlm.nih.gov/nucleotide/MK870785.1?report=genbank&log$=nucltop&blast_rank=2&RID=FFYD4KZ7016) |
| [Trichoderma sp. isolate SDAS203090 small subunit ribosomal RNA gene, partial sequence; internal transcribed spacer 1, 5.8S ribosomal RNA gene, and internal transcribed spacer 2, complete sequence; and large subunit ribosomal RNA gene, partial sequence](https://blast.ncbi.nlm.nih.gov/Blast.cgi#alnHdr_1628768451) | 1098 | 1098 | 100% | 0.0 | 100.00% | [MK870384.1](https://www.ncbi.nlm.nih.gov/nucleotide/MK870384.1?report=genbank&log$=nucltop&blast_rank=3&RID=FFYD4KZ7016) |
| [Trichoderma sp. PPRI 10262 strain PPRI_10262 internal transcribed spacer 1, partial sequence; 5.8S ribosomal RNA gene and internal transcribed spacer 2, complete sequence; and large subunit ribosomal RNA gene, partial sequence](https://blast.ncbi.nlm.nih.gov/Blast.cgi#alnHdr_1178873539) | 1098 | 1098 | 100% | 0.0 | 100.00% | [KY069815.1](https://www.ncbi.nlm.nih.gov/nucleotide/KY069815.1?report=genbank&log$=nucltop&blast_rank=4&RID=FFYD4KZ7016) |
| [Trichoderma sp. isolate 41 small subunit ribosomal RNA gene, partial sequence; internal transcribed spacer 1, 5.8S ribosomal RNA gene, and internal transcribed spacer 2, complete sequence; and large subunit ribosomal RNA gene, partial sequence](https://blast.ncbi.nlm.nih.gov/Blast.cgi#alnHdr_1508178762) | 1098 | 1098 | 100% | 0.0 | 100.00% | [MK120584.1](https://www.ncbi.nlm.nih.gov/nucleotide/MK120584.1?report=genbank&log$=nucltop&blast_rank=5&RID=FFYD4KZ7016) |
| [Fungal sp. strain UBDFT20 small subunit ribosomal RNA gene, partial sequence; internal transcribed spacer 1, 5.8S ribosomal RNA gene, and internal transcribed spacer 2, complete sequence; and large subunit ribosomal RNA gene, partial sequence](https://blast.ncbi.nlm.nih.gov/Blast.cgi#alnHdr_1502246254) | 1098 | 1098 | 100% | 0.0 | 100.00% | [MK116425.1](https://www.ncbi.nlm.nih.gov/nucleotide/MK116425.1?report=genbank&log$=nucltop&blast_rank=6&RID=FFYD4KZ7016) |
| [Fungal sp. strain UBDFT19 small subunit ribosomal RNA gene, partial sequence; internal transcribed spacer 1, 5.8S ribosomal RNA gene, and internal transcribed spacer 2, complete sequence; and large subunit ribosomal RNA gene, partial sequence](https://blast.ncbi.nlm.nih.gov/Blast.cgi#alnHdr_1502246252) | 1098 | 1098 | 100% | 0.0 | 100.00% | [MK116424.1](https://www.ncbi.nlm.nih.gov/nucleotide/MK116424.1?report=genbank&log$=nucltop&blast_rank=7&RID=FFYD4KZ7016) |
| [Trichoderma harzianum strain NECC30402 small subunit ribosomal RNA gene, partial sequence; internal transcribed spacer 1, 5.8S ribosomal RNA gene, and internal transcribed spacer 2, complete sequence; and large subunit ribosomal RNA gene, partial sequence](https://blast.ncbi.nlm.nih.gov/Blast.cgi#alnHdr_1487130141) | 1098 | 1098 | 100% | 0.0 | 100.00% | [MH153633.1](https://www.ncbi.nlm.nih.gov/nucleotide/MH153633.1?report=genbank&log$=nucltop&blast_rank=8&RID=FFYD4KZ7016) |
| [Trichoderma sp. isolate yi1211_1 small subunit ribosomal RNA gene, partial sequence; internal transcribed spacer 1, 5.8S ribosomal RNA gene, and internal transcribed spacer 2, complete sequence; and large subunit ribosomal RNA gene, partial sequence](https://blast.ncbi.nlm.nih.gov/Blast.cgi#alnHdr_1386677565) | 1098 | 1098 | 100% | 0.0 | 100.00% | [MH284652.1](https://www.ncbi.nlm.nih.gov/nucleotide/MH284652.1?report=genbank&log$=nucltop&blast_rank=9&RID=FFYD4KZ7016) |
| [Trichoderma sp. isolate yi0734_1 small subunit ribosomal RNA gene, partial sequence; internal transcribed spacer 1, 5.8S ribosomal RNA gene, and internal transcribed spacer 2, complete sequence; and large subunit ribosomal RNA gene, partial sequence](https://blast.ncbi.nlm.nih.gov/Blast.cgi#alnHdr_1386677125) | 1098 | 1098 | 100% | 0.0 | 100.00% | [MH284212.1](https://www.ncbi.nlm.nih.gov/nucleotide/MH284212.1?report=genbank&log$=nucltop&blast_rank=10&RID=FFYD4KZ7016) |

The strain was identified as *Trichoderma sp*. and suspected to be *Trichoderma harzianum*.


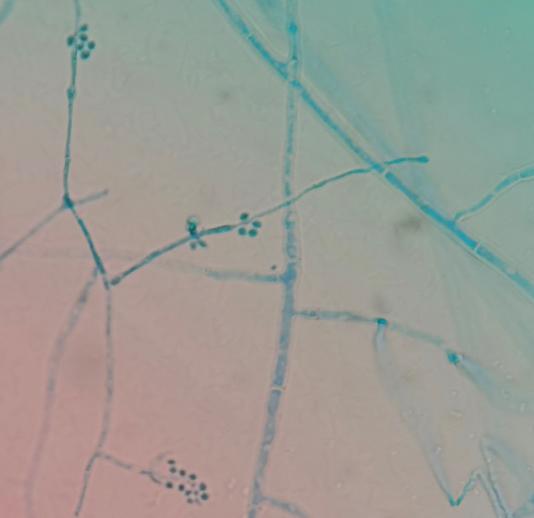

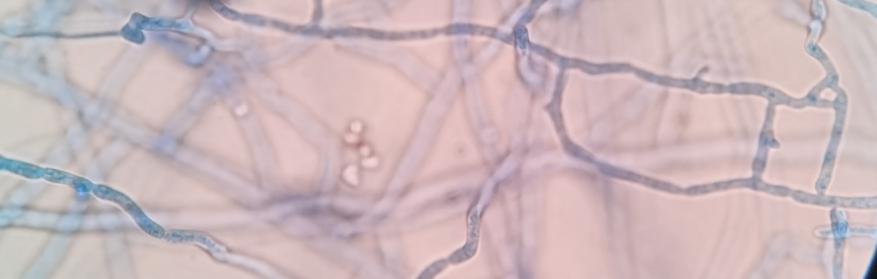

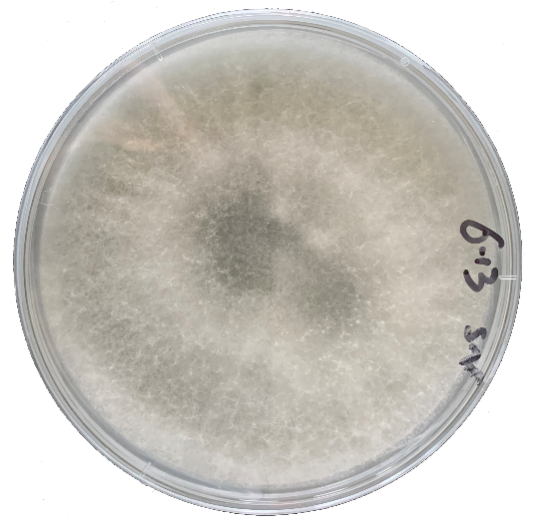


**Fig.1** Morphological characteristics and spore morphology of T *Trichoderma harzianum* observed under microscope (OLYMPUS CX21FS1, 400×).

**2. *T. harzianum* isolate identification and sequencing**

(1) DNA extraction

The genome was extracted by DNA extraction kit. DNA quality detection: 1. take 5 μ L DNA solution 1% agarose, 1 × TAE buffer electrophoresis (voltage 120-180 V) detection, a single band shows that DNA complete no degradation, there are obvious bands indicating that the concentration and purity can meet the requirements of PCR; 2. spectrophotometer detection concentration and purity, take 1 μ L OD value, OD260/280 is 1.7 μ L 2.0, indicating that DNA quality is good, less than 1.7 protein pollution, more than 2.0 RNA pollution. Generally, a small amount of protein and RNA pollution does not affect ordinary PCR.

(2) PCR reaction system

| Template DNA | 20～50 ng/μL | 1～2μL |
| --- | --- | --- |
| Primer F | 10μM | 2μL |
| Primer R | 10μM | 2μL |
| dNTP (mix) | 10mM | 2μL |
| Taq Buffer (with MgCl_2_) | 10x | 5μL |
| Taq Mix | 5 U/μL | 0.5μL |

(3) PCR reaction conditions

| Serial number | Program | Temperature | Time |
| --- | --- | --- | --- |
| 1 | Pre-denaturation | 95℃ | 3min |
| 2 | Denaturation | 94℃ | 30sec |
| 3 | Annealing | 55-60℃ | 30sec |
| 4 | Extend | 72℃ | 50sec |
| 5 | Cycle 2 to 4 | 35cycles | |
| 6 | Repair and extension | 72 | 10min |

(4) Electrophoresis detection of bands

The PCR products were taken from 5 μL 1% agarose gel electrophoresis, and the electrophoresis parameters were 150V, 100mA, 10min and 20min (see electrophoretic map).


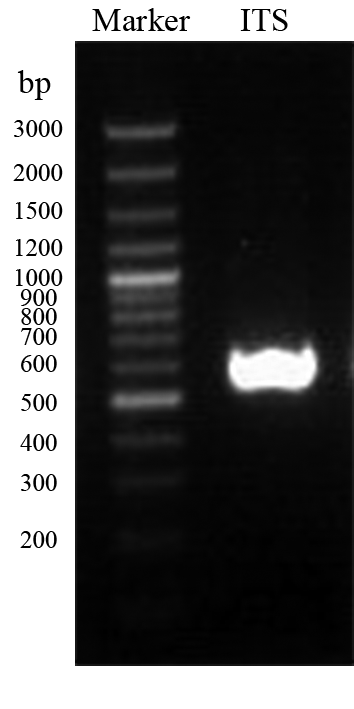


(5) Sequencing

The PCR product was purified back. Objective PCR band cut glue was recovered. The method can be seen in the PCR product purification recovery kit.

Sequencing of PCR products PCR reaction, standard reaction system:

| Purified PCR product | 10 ng /μL | 1μL |
| --- | --- | --- |
| BigDye | 2.5× | 4μL |
| BigDye Seq Buffer | 5× | 2μL |
| Sequencing primer | 3.2 pmol /μL | 1μL |
| Sterilized deionized water | / | 12μL |
| Total volume | / | 20μL |

PCR sequencing reaction parameters

| Serial number | Program | Temperature | Time |
| --- | --- | --- | --- |
| 1 | Pre-denaturation | 96℃ | 1min |
| 2 | Denaturation | 96℃ | 10sec |
| 3 | Annealing | 50℃ | 5sec |
| 4 | Extend | 60℃ | 4min |
| 5 | Cycle 2 to 4 | 25cycles | |
| 6 | Stop heat preservation | 4℃ | ∽ |
