# Supplementary figures and images for: Characterization of a novel recombinant halophilic β-glucosidase of Trichoderma harzianum derived from Hainan mangrove
Source: BMC Microbiol. 2022 Jul 28;22:185. doi: 10.1186/s12866-022-02596-w (PMC9331182; doi:10.1186/s12866-022-02596-w)

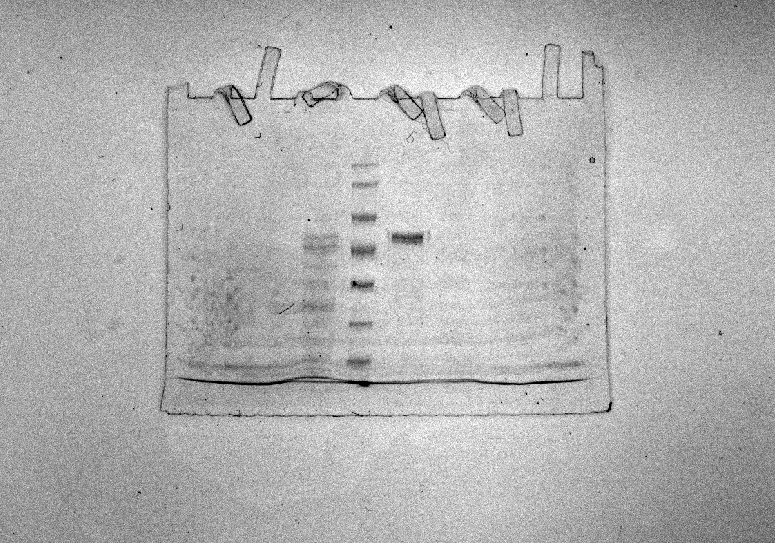

Supplement: Supplementary file 4 — Additional file 4. [file 12866_2022_2596_MOESM4_ESM.jpg]
